# Severe neurological sequelae and behaviour problems after cerebral malaria in Ugandan children

**DOI:** 10.1186/1756-0500-3-104

**Published:** 2010-04-16

**Authors:** Richard Idro, Angelina Kakooza-Mwesige, Stephen Balyejjussa, Grace Mirembe, Christine Mugasha, Joshua Tugumisirize, Justus Byarugaba

**Affiliations:** 1 Department of Paediatrics and Child Health, Mulago Hospital and Makerere University School of Medicine, PO Box 7072, Kampala, Uganda; 2 Department of Psychiatry, Mulago Hospital and Makerere University School of Medicine, PO Box 7072, Kampala, Uganda

## Abstract

**Background:**

Cerebral malaria is the most severe neurological complication of falciparum malaria and a leading cause of death and neuro-disability in sub-Saharan Africa. This study aimed to describe functional deficits and behaviour problems in children who survived cerebral malaria with severe neurological sequelae and identify patterns of brain injury.

**Findings:**

Records of children attending a specialist child neurology clinic in Uganda with severe neurological sequelae following cerebral malaria between January 2007 and December 2008 were examined to describe deficits in gross motor function, speech, vision and hearing, behaviour problems or epilepsy. Deficits were classified according to the time of development and whether their distribution suggested a focal or generalized injury. Any resolution during the observation period was also documented.

Thirty children with probable exposure to cerebral malaria attended the clinic. Referral information was inadequate to exclude other diagnoses in 7 children and these were excluded. In the remaining 23 patients, the commonest severe deficits were spastic motor weakness (14), loss of speech (14), hearing deficit (9), behaviour problems (11), epilepsy (12), blindness (12) and severe cognitive impairment (9). Behaviour problems included hyperactivity, impulsiveness and inattentiveness as in attention deficit hyperactivity disorder (ADHD) and conduct disorders with aggressive, self injurious or destructive behaviour. Two patterns were observed; a) immediate onset deficits present on discharge and b) late onset deficits. Some deficits e.g. blindness, resolved within 6 months while others e.g. speech, showed little improvement over the 6-months follow-up.

**Conclusions:**

In addition to previously described neurological and cognitive sequelae, severe behaviour problems may follow cerebral malaria in children. The observed differences in patterns of sequelae may be due to different pathogenic mechanisms, brain regions affected or extent of injury. Cerebral malaria may be used as a new model to study the pathogenesis of ADHD.

## Background

Cerebral malaria is the most severe neurological complication of falciparum malaria. Children with cerebral malaria are admitted with fever, seizures, coma and brainstem signs and despite adequate treatment using current guidelines, about 20% die[1]. Earlier studies suggested that there is full neurological recovery[2] but over the past 15 years, it has become increasingly clear that many children sustain severe brain injury after cerebral malaria and 25% have long-term neurological and cognitive deficits or epilepsy [3-8]. Thus, cerebral malaria is now considered a leading cause of neuro-disability in sub-Saharan Africa[9].

A wide range of functional deficits are described [3-7], the incidence of some of which increase with time[5]. However, the understanding of the pathogenesis of brain injury is poor. Observational studies have identified repeated and prolonged seizures, intracranial hypertension, severe metabolic derangement, deep and prolonged coma as risk factors for poor outcome [10-13]. However, how these factors interact to cause neural injury is unknown. Recognition of the pattern of functional deficits in surviving children may help in understanding the pathogenesis of neural injury. In this study, we examined records of children who survived cerebral malaria with severe neurological impairment, described their functional deficits and identified patterns of brain injury.

## Methods

### Design

This was a case series of children exposed to cerebral malaria that developed severe neurological deficits and attended specialist neurology care.

### Setting

The weekly Child Neurology Clinic in Mulago (the teaching hospital for Makerere University School of Medicine) in Kampala, Uganda, is the only specialist care centre for children with neurological disorders in the country[14]. This outpatient clinic is run by one paediatric neurologist and two specialist registrars and although there has been some improvement in recent times, the clinic is unable to offer comprehensive care or rehabilitation. Electromyography, laboratory testing into the metabolic causes of neurological disease, genetic testing and counselling and Magnetic Resonance Imaging is unavailable. The clinic attends to children from all over the country but access is limited by geographical and economic factors, local beliefs on the causation of neurological illness and the influence of traditional medical practices.

### Subjects

Study subjects were children ages 12-79 months, who attended the clinic from January 2007 to December 2008;

a) following hospitalization for World Health Organization defined cerebral malaria (unarousable coma with *Plasmodium falciparum *parasites on a blood smear, a normal cerebrospinal fluid and no other cause to explain the coma)[15],

b) were discharged with or had severe neurological sequelae developing after discharge and

c) had detailed referral information, a discharge summary or both.

All had been exposed to cerebral malaria between the ages of 5-46 months and first attended the clinic 1-48 months later. Subsequent re-attendances were at intervals determined by the attending clinician but mostly at 1-3 month intervals and each subject was followed up for a minimum of six months. Children with incomplete records or for whom the reported diagnosis of cerebral malaria could not be ascertained were excluded.

### Procedures

Records of incident patients who attended the clinic during the study period and fulfilled the inclusion criteria were examined. Parental reports of acquired deficits in vision, hearing or speech, regression in milestones and severe cognitive impairment, gross motor deficits and severe behavioural problems were documented. Motor assessment was based on a system for classifying function in children with cerebral palsy, the gross motor function classification system (GMCFS)[16]. This is a five-level grading of motor deficits and a hierarchical classification of spasticity and ataxia. Epilepsy was defined as two or more seizures unrelated to fever following exposure to cerebral malaria[5] and was classified with EEG testing. Because the children came from different cultures - the majority of which do not have any adaptation of IQ tests - we were unable to conduct formal IQ testing but only recognised severe cognitive impairment based on the child's abilities in the areas of play, communication, safety, self-care and/or school as is expected for age. Thus, severe cognitive impairment was documented if there was severe impairment in adaptive functioning in play, communication, safety, self-care and/or school as is expected for age. This was however further modified for children 1-3 years old in whom the ability to engage in imaginative play was the main domain examined. Play was described by the parent and observed in the clinic and scored dichotomously as severely impaired or not. Severe neurological sequelae was defined as any or a combination of severe motor deficit of grades III-V on the GMCFS, blindness, severe hearing or speech impairment, epilepsy, behaviour problems and cognitive impairment that impaired daily activities or play. HIV testing and neuro-imaging was not routinely available although in a related study, only 3% of children with epilepsy in the clinic in 2009 were HIV infected (Christine Mugasha, unpublished). Specific therapy e.g. anti epileptic drugs and rehabilitation were offered within available means. Visual and hearing assessments were often limited by the cognitive function of the children. The study was approved by Makerere University School of Medicine as part of a project to describe neurological disorders in Ugandan children.

## Results

### General description

Of the 1,136 incident patients registered in the clinic over the two-year period, 835 were attended to and had records available for review. The majority of missing records were for patients who were registered in the clinic, had an appointment booked but did not attend. Thirty patients out of the 835 reported exposure to cerebral malaria of whom, 23 fulfilled the inclusion criteria. Original hospitalisation notes were obtained for 11 of these patients. Seven patients did not have sufficient information to exclude other central nervous system infections. The median age on exposure was 30 (5-72) months and 13 were males.

### Types of severe sequelae

**Figure 1:**
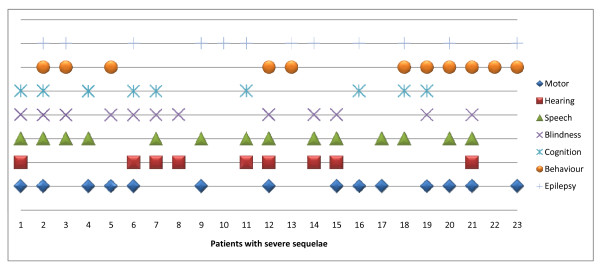
**The types and distribution of severe sequelae following cerebral malaria**. Figure 1 describes severe sequelae and impairments in different domains (motor, speech, hearing, vision and cognition or epilepsy) in 23 individual children. Multiple sequelae were common.

The majority of patients had been discharged with multiple deficits. Severe neurological sequelae included loss of speech (14), hearing impairment (9), motor deficits (14), behaviour problems (11), epilepsy (12), blindness (12) and cognitive impairment (9). Figure 1 is a summary of the distribution of sequelae in individual patients.

#### a) Pattern of neurological sequelae

Based on the time of onset, severe neurological sequelae could be categorized into two;

i. Immediate onset sequelae - evident on discharge from hospital. The majority of these patients had prolonged coma and status epilepticus during the acute illness (Additional file 1). Again, two forms were recognisable:

a. Focal sequelae such as hemiplegia and focal seizures.

b. Multifocal or generalized sequelae with spastic quadriparesis, movement disorders, cognitive impairment, blindness, loss of social skills, speech or hearing impairment.

ii. Late onset sequelae - developed within months (behaviour problems) or months to years (epilepsy) after the insult.

#### b) Specific types of sequelae

##### Blindness

Cortical blindness has been described in earlier studies and because it is reversible, its pathogenesis has been attributed to transient ischaemia. In this series, blindness was observed in 12/23 subjects and often, was one of many deficits on discharge. The shortest time to resolution was one month. Visual acuity in ten of the remaining patients improved over a 2-6 months period and in all cases, almost normal vision was restored by 7 months.

##### Hearing impairment

Hearing impairment has rarely been reported in previous series. In this series, severe hearing impairment was reported in 9/23 children. Two other children had milder degrees of hearing impairment. Five of the nine children with severe impairment had formal audiometric testing: 3 had profound impairment and 2 had severe impairment with a hearing threshold of 80 decibels. The parents of only one child could afford hearing aids.

##### Loss of speech

Aphasia was reported in 14 children. Three other children had dysarthria. All three had oro-motor problems. Although speech therapy was offered at weekly or fortnightly intervals, over the follow up period, no full recovery was reported suggesting that loss of speech after childhood cerebral malaria has a poorer prognosis.

##### Gross motor deficits

Gross motor deficits following cerebral malaria include hemiplegia, diplegia, quadriparesis or quadriplegia[17]. In this study, severe motor deficits of GMFCS grades III-V were reported in 14/23 children. Six of the fourteen children also had movement and gait disorders (ataxia, choreoathetosis, dystonia and poor neck control). Motor deficits were aggravated by concurrent movement and gait problems and associated with regression in milestones and feeding difficulties (some previously ambulant children were discharged unable to sit unsupported or walk). Function however improved with physiotherapy and resolving ataxia.

##### Behaviour problems

Behaviour problems observed in 11 children were some of the most striking difficulties. Using items in the DSM IV-TR criteria, 3 types of behaviour problems could be recognized:

a) Hyperactivity, impulsiveness and inattentiveness as in ADHD;

b) Conduct disorders (aggressive, destructive and obsessive behaviour);

c) Autistic spectrum and pervasive developmental disorder-like behaviours with or without other neuro-psychiatric problems (eating rubbish, running away from home and self injurious behaviour).

The earliest behavioural symptoms developed within two weeks of discharge. The most common were ADHD-like symptoms. These often were associated with poor night-time sleep. Symptoms improved over 1-3 weeks with haloperidol and/or small doses of methylphenidate. Worsening hyperactivity developed in a child with self injurious behaviour following the initiation of phenobarbitone to treat epilepsy. Behaviour problems in the 12^th ^child were presumed to be an adverse reaction to phenobarbitone and the symptoms improved when this drug was withdrawn.

Aggressive behaviour included beating up peers, throwing stones at people and cars with no or minimal provocation and in two cases, uncontrollable anger. One child with gelastic seizures and a history of febrile seizures in multiple family relations would in addition to anger and aggressive behaviour get prolonged bouts of laughter.

Parents of two other children with labile moods reported bouts of excessive crying following minimal scolding. In the first of the two children, these crying episodes started two weeks after discharge from hospital; the child would cry continuously for over two hours. The electroencephalogram was normal.

Three other children were said to wander off and would sometimes get lost unless closely watched. Parents of one would tie him up in the house. The strain on the family was so immense that one parent described life at home almost like, "hell" and grandparents were called in to help care for two of the other children. These symptoms appeared to improve with haloperidol: in 2/3 cases (including the child who was being tied up), the wandering stopped within a month.

Other than socialization, the effect of the behaviour problems on school attendance in previously school-going children was detrimental; teachers could not cope, school performance declined significantly and all dropped out of school. A brain CT scan was performed in two children - one, a child with intractable epilepsy and aggressive behaviour who was initially discharged with blindness, loss of hearing and speech. The scans showed no focal lesions in the brain parenchyma but bilateral atrophy of the temporal lobes. The second CT scan was in a child discharged with hemiplegia, blindness, feeding difficulties, loss of speech and who later developed ADHD-like symptoms. The acute CT showed brain swelling and meningeal enhancement over the temporo-parietal area and the convalescent CT, cortical atrophy in the same area.

##### Epilepsy

EEG was performed in 8 of the 12 patients with epilepsy. In patients with generalized seizures, diffuse epileptiform discharges were observed on the electroencephalogram. Localized discharges over the temporal region were seen in two patients. Two children had multiple seizure types with more than 10 seizures a day. Seizures in these two were unresponsive to therapy with phenobarbitone (later withdrawn) and carbamazepine. Although seizures in one eventually improved on sodium valproate, in the second child who in addition had severe behavioural problems, sodium valproate did not reduce the seizure frequency. Instead, the frequency reduced and the behaviour problems improved upon the initiation of clonazepam.

##### Other problems

Several patients with multiple sequelae had severe cognitive impairment. Child neglect and malnutrition were evident especially among those who presented >6 months after exposure. Malnutrition was compounded by feeding difficulties in the spastic children.

## Discussion

This study aimed to describe functional and behaviour deficits in children with severe impairments following cerebral malaria and the patterns of these deficits. We found that deficits in motor function, behaviour, vision, speech and hearing or epilepsy were major long-term sequelae. Two main patterns were observed: a) immediate and, b) late-onset deficits. Some deficits (e.g. blindness) resolved, others (e.g. loss of speech) showed little improvement over the follow-up period, while some (e.g. behaviour problems) developed long after exposure.

Earlier studies of cerebral malaria mostly focused on reducing the high mortality. Although the long-term outcome has been described in recent times[4,6,7,12] and some behaviour problems were mentioned in some studies[8,18], the current series is the first fairly detailed description of the behaviour problems after childhood cerebral malaria. The pathogenesis is unclear but hypoxic neural injury, secondary to parasite sequestration in the cerebral capillary bed during the acute disease, may play a part. Our study also describes hearing deficits after cerebral malaria and the poorer prognosis of some sequelae such as loss of speech. Thus, the effects of cerebral malaria are beyond the deaths it is reputed to cause; it may be responsible for impairments in hundreds of thousands of children in the malaria-endemic areas of sub-Saharan Africa. Many of these children may be missing out on school with enormous socio-economic consequences. The stress of managing children with severe impairment and behaviour difficulties may also be putting a lot of strain on family relationships. Strategies for follow-up and continuing care, rehabilitation and family counselling should therefore be developed and incorporated into care protocols. A recent pilot study to rehabilitate children with cognitive deficits after cerebral malaria showed encouraging results with improvements in cognitive function and behaviour[19]. There is also need for funding rehabilitation services and the provision of assistive devices for the majority of families unable to foot such bills. For example, the family of only one child out of nine with severe to profound hearing impairment in this series could afford the cost of hearing aids for their child.

This was a retrospective study of children with severe impairments in a specialist clinic but not a representative sample of children surviving cerebral malaria, a selection bias that may have over-estimated the frequency of sequelae. Secondly, the World Health Organization definition for cerebral malaria is not specific[20] and so, some of these sequelae may have followed other encephalopathies with malaria parasitaemia only an incidental finding. Third, we neither assessed the effects of HIV infection nor could we ascribe hearing impairment solely to cerebral malaria since quinine, the anti malaria drug of choice, can cause similar impairment[21]. Fourth, although the subjects were systematically assessed, data collection was not designed to obtain data on specific entities such as the autistic spectrum disorders and other neuro-psychiatric disorders. Prospective studies that have malaria retinopathy as a discriminating sign are needed to clearly define these problems[22]. Preferably, such studies should include incidence, biomarkers of brain injury and neuro-imaging to study pathogenesis.

Despite the above weaknesses and the small sample size, this is one of the first papers to provide a comprehensive description of impairments following recovery from cerebral malaria by using domain specific assessments in individual children. The inclusion of behaviour assessments has for the first time highlighted behaviour difficulties as major sequelae of cerebral malaria in children. In addition, the two patterns of deficits described here may help guide pathogenesis studies although the observed differences may be due to different brain regions affected or extent of injury. Cerebral malaria may also be used as a new model to study the pathogenesis of ADHD in children.

## Competing interests

The authors declare that they have no competing interests.

## Authors' contributions

RI and TJ conceived the study. RI, AK, SB, GM, CM and JB saw the patients. RI performed the analysis and wrote the first draft while AK SB, JB and TJ critically read through the manuscript.

## Supplementary Material

Additional file 1 **Supplementary Table S1: Severe Neurological and Behavioural Sequelae following Cerebral Malaria in Ugandan children**. This table summarises the demographic and clinical characteristics of the individual study subjects at the time of exposure, the documented risk factors for neurological sequelae, the types of sequelae observed and changes over the follow up period. Click here for file
