# Supplementary material for: Severe neurological sequelae and behaviour problems after cerebral malaria in Ugandan children
Source: BMC Res Notes. 2010 Apr 16;3:104. doi: 10.1186/1756-0500-3-104 (PMC2861066; doi:10.1186/1756-0500-3-104)
Supplement: Additional file 1 — Supplementary Table S1: Severe Neurological and Behavioural Sequelae following Cerebral Malaria in Ugandan children. This table summarises the demographic and clinical characteristics of the individual study subjects at the time of exposure, the documented risk factors for neurological sequelae, the types of sequelae observed and changes over the follow up period. [file 1756-0500-3-104-S1.DOC]

**Supplemental Table 1**

**Severe Neurological and Behavioural Sequelae following Cerebral Malaria in Ugandan children**

| **ID No.** | **Age on exposure, months** | **Gender** | **Time from exposure to attending clinic, months** | **Risk factors for poor outcome** | **Time from discharge to sequelae** | **Types of sequelae observed** | **Changes on EEG** | **Comments** |
| --- | --- | --- | --- | --- | --- | --- | --- | --- |
| 1 | 24 | Male | 1 | Status epilepticus | Immediate. Discharged with multiple deficits. | 1. Quadriparesis with inability to feed self 2. Severe cognitive impairment 3. Loss of hearing 4. Loss of speech 5. Blindness | N/A | Lost to follow up. |
| 2 | 30 | Male | 18 | Severe anaemia  Status epilepticus | Immediate. Discharged with multiple deficits. | 1. Loss of speech 2. Severe visual impairment – only able to perceive light 3. Quadriparesis 4. Severe cognitive impairment with loss of toilet control. 5. Behaviour problems with hyperactivity, aggression impulsiveness. 6. Focal epilepsy initially had non convulsive seizures (startles) | Sleep EEG shows epileptic discharges from both temporal lobes. Left focus has tendency to generalization | Recovered vision. |
| 3 | 72 | Male | 7 | Status epilepticus  Deep and prolonged coma | Immediate. Discharged with multiple deficits. | 1. Blindness 2. Severe hearing impairment 3. Loss of speech 4. Chorea 5. Generalized tonic clonic epilepsy 6. Hyperactivity and short attention span | EEG testing not done. | Vision improved over 7 months.  Refractory seizures despite anticonvulsants. |
| 4 | 23 | Female | 1 | Status epilepticus Deep and prolonged coma | Immediate. Discharged with multiple deficits. | 1. Quadriparesis 2. Loss of speech 3. Impaired hearing 4. Severe cognitive impairment, loss of social skills including independent feeding and toilet training | N/A | No improvement |
| 5 | 30 | Male | 1 | Not reported | Immediate.  Discharged with multiple deficits. | 1. Quadriparesis 2. Severe visual impairment 3. Disordered breathing and drooling 4. Severe hyperactivity | N/A | Later, a diagnosis of ADHD was made. |
| 6 | 17 | Female | 0.75 | Not reported | Immediate.  Discharged with multiple deficits. | 1. Loss of hearing 2. Blindness 3. Quadriplegia 4. Choreoathetosis 5. Severe cognitive impairment 6. Epilepsy | Low amplitude activity. No epileptiform discharges. | Seizure activity associated with sudden onset of limb movements followed by deep guttural sounds. |
| 7 | 46 | Female | 4 | Deep and prolonged | Immediate.  Discharged with multiple deficits. | 1. Loss of speech 2. Loss of hearing 3. Blindness 4. Alternating limb tone and choreoathetosis 5. Severe cognitive impairment | N/A | Developed severe choreoathrtoid movement disorder with fluctuating tone 1 month after exposure. |
| 8 | 11 | Female | 1 | Not reported | Immediate.  Discharged with multiple deficits. | 1. Blindness 2. Loss of hearing 3. Movement disorder | N/A | . Blindness resolved. |
| 9 | 5 | Male | 25 | Hypoglycaemia  Deep coma  Status epilepticus | Immediate.  Discharged with multiple deficits. | 1. Loss of speech 2. Quadriparesis 3. Epilepsy | Diffuse epileptiform discharges | Only minimal improvement in motor function |
| 10 | 24 | Male | 4 years | Status epilepticus | 4 years | 1. Epilepsy. Doing very well in School (most recent position in class, 1^st^ out of 49). | EEG testing not done. | On treatment with Phenobarbitone. |
| 11 | 40 | Male | 1 month | Status epilepticus | Immediate. Discharged with multiple deficits | 1. Loss of speech 2. Severe hearing impairment 3. Epilepsy 4. Severe cognitive impairment | Mild diffuse epileptiform activity with no focus. | On treatment with Phenobarbitone and has hearing aids. |
| 12 | 17 | Male | 4 months | Severe anaemia  Hypoglycaemia  Refractory status epilepticus | Immediate. Discharged with multiple deficits | 1. Blindness 2. Hearing loss 3. Loss of speech 4. Left sided hemiplegia (worst in upper limbs) 5. Hyperactive and short attention span | N/A | Regained sight over 6 weeks and had partial improvement in hearing over 7 weeks. Difficulty in chewing. |
| 13 | 23 | Male | 5 months | 1. Prolonged coma | Some immediate others 2 months | 1. Epilepsy 2. Progressively worsening behaviour problems (inattentive, hyperactive and aggressive) 3. Un-coordinated speech | Mild diffuse epileptiform activity | Seizures controlled with carbamazepine. Aggressiveness and hyperactivity are major problems. |
| 14 | 31 | Male | - 1. months | Status epilepticus  Deep and prolonged coma | Immediate.  Discharged with multiple deficits. | 1. Blindness 2. Loss of hearing 3. Loss of speech 4. Intractable epilepsy 5. Behaviour problems (excessive movements, aggression, shouting, removes clothing) | Diffuse slow background and spike wave discharges similar to EEG in Lennox Gastaut epilepsy | Initially on Phenobarbital but withdrawn after developing hyperactivity. Seizures failed to respond to carbamazepine and sodium valproate. Both seizures and hyperactivity reduced on clonazepam. |
| 15 | 30 | Male | 0.75 (3 weeks) | Not reported | Immediate. Discharged with multiple deficits. | 1. Quadriplegia with   Inability to sit unsupported and had feeding problems   1. Blindness 2. Loss of speech 3. Impaired hearing | N/A | Vision improved to perceiving light in 2 months and near normal in 6months. He was able to walk alone within 5 months. Incomprehensible speech 6 months after exposure. On regular speech therapy. |
| 16 | 30 | Female | 12 | Status epilepticus | Immediate. Discharged with multiple deficits. | 1. Epilepsy, multiple seizure types, 10-15 seizures a day. 2. Quadriparesis 3. Severe cognitive impairment and regression in milestones with severe seizures. | Diffuse slow waves and focal discharges over right posterior temporal region. | Poor seizure response to Phenobarbital. Improved on sodium valproate. |
| 17 | 13 | Female | 3 | Hypoglycaemia | Some immediate others 1 month later. | 1. Quadriparesis with inability to sit 2. Choreoathetoid movements 3. Blindness | N/A | Vision started to improve in 2 months and motor deficits within 3 months. |
| 18 | 24 | Female | 48 | Prolonged coma | Immediate. Discharged with multiple deficits. | 1. Loss of speech 2. Severe behaviour problems (destructive, excessive anger, injures self when angry, hyperactive and in attentive). Has periods when she eats rubbish and runs away from home. 3. Severe cognitive impairment and learning disability 4. Epilepsy | EEG testing not done. | Epileptic seizures controlled with carbamazepine. |
| 19 | 25 | Female | 54 | Multiple risk factors | Immediate. Discharged with multiple deficits. | 1. Incomprehensible speech 2. Quadriplegia 3. Severe cognitive impairment with loss of toilet control 4. Blindness 5. Severe behaviour and psychiatric problems (biting, eats rubbish, chews clothing, hyperactivity and in attentiveness) | N/A | Regained vision after 1 mo and walked 4 yrs later. The behaviour problems progressively worsened over the 4 years. Given haloperidol and methylphenidate. Lost only sibling to cerebral malaria. Ten yr old cousin has severe sequelae after cerebral malaria. |
| 20 | 31 | Male | 78 | Status epilepticus  Prolonged coma | Immediate. Discharged with multiple deficits. | 1. Loss of speech 2. Right sided hemiplegia with cranial nerves VI and VII palsies 3. Secondarily generalized epilepsy. 4. Self injurious behaviour, (bites himself), shouts and makes noises. 5. Sleep disorder – poor sleep | EEG testing not done | No family history of neurological disorders. Seizures frequency reduced on Phenobarbital but behaviour problems worsened. Phenobarbital replaced with Carbamazepine. |
| 21 | 17 | Male | 48 | Status epilepticus  Hypoglycemia  Prolonged coma | Immediate. Discharged with multiple deficits. | 1. Blindness 2. Loss of speech 3. Impaired hearing 4. Behaviour problems (hyperactive and short attention span). 5. Left sided hemiplegia 6. Feeding difficulties. | N/A | Regained vision and hearing 3 and ½ months after discharge. |
| 22 | 49 | Female | 0.5 (2 weeks) | Repeated focal seizures after initial improvement | One week. | 1. Excessive anger and episodes of excessive happiness/laughter. Probable gelastic seizures. 2. Aggressive behaviour | EEG testing not done | Multiple family members reporting febrile seizures. Lost to follow up. |
| 23 | 37 | Female | 12 | Refractory status epilepticus  Prolonged coma | Immediate.  Discharged with multiple deficits. | 1. Left sided hemiplegia and VII palsy. 2. Slurred speech 3. Ataxia 4. Epilepsy 1 month after discharge 5. Hyperactivity | Generalized sharp waves with diffuse epileptiform activity. No focus | Seizures controlled and patient improved on Carbamazepine. |
